# The Treatment of Rheumatism with Citric Acid

**Published:** 1853-12

**Authors:** S. A. Ogier


### *On the Treatment of Rheumatism with Citric acid.*

BY S. A. OGIER, M. D.

From the recommendation of some of the European journals, of lemon juice in the treatment of Rheumatism, I was induced to commence a trial of it about two years since, and on the whole the results have been satisfactory.

I have selected the two following cases as striking illustrations of the beneficial influence of the citric acid treatment, at the same time I must say, that in many cases the results, though favorable, were not marked with such prompt relief.

Case 1st. T. N., aged 50, of robust constitution was attacked with Rheumatism on 20th of June, '52. On the 22d I was called to see him, and found him with a hot skin, pulse frequent—120 beats to the minute—he was unable to move in bed, all the joints affected, the tongue presented a thick brown coat, though moist. I prescribed a brisk cathartic to be followed by a full dose of Pulv. Doveri at night; the joints to be rubbed and swathed with turpentine liniment. 23d.—I found him about the same; the cathartic had moved the bowels freely, the tongue less coated. I now put him on the use of the wine of Colchicum, with cups to the back, cathartics and full doses of opii., counter-irritating and narcotic liniments and V. S. This plan of treatment was continued for a week without any decided relief. On the 30th my patient having become much reduced, and the opium &c. failing to afford him relief, I put him on the liberal use of Cit. Acid. I used the crystalized acid in place of lemon juice which I could not procure, making a solution of the former of the officinal strength of the lemon juice; of this half an ounce was directed to be taken in a little sugar and water every 4 hours. Under this treatment, my patient rapidly recovered, the secretion of urine was greatly increased, the fever abated, and at the end of the 4th day from the commencement of the use of the acid, he was able to walk about with the aid of a cane. The effect of the acid upon the pulse was remarkable; from 120 it was reduced to 74 in the minute. This effect upon the action of the heart has been noticed by Dr. G. O. Rees in the 12th No. of Ranking's Abstract for 1850. In numerous other cases I have myself witnessed it.

Case No. 2. Wm. S., aged 22. I was requested to see this young man on the night of the 1st of December. I found him in great pain from an attack of Rheumatism which had come on two days previous to my visit. He had but a few months recovered from an attack that had confined him to the house for several weeks. He now had a hot skin, with some fever, the joints a little swollen and acutely painful, tongue much coated, urine scanty and high colored. Not having the acid with me, I temporised until morning. On the 2d.—I found him about the same as the previous night, the bowels had been freely moved by a cathartic prescribed at that time. He had not been able to sleep.

I ordered the Cit. Acid solution as in the former case with Pulv. Doveri, grs. viii. to be taken at night. Dec. 3d. he had less fever, got a little sleep, joints still very painful, urine about as yesterday, no appetite. Continued treatment.

4th. Passed a better night, pulse much less frequent, skin cooler, urine abundant and lighter colored, joints still painful.

5th. Found my patient sitting up in his chamber eating breakfast, the pulse natural, tongue clean, urine abundant, joints much less painful.

6th. To-day I found my patient in the parlor, protesting against any more medicine, though delighted with the prompt relief it had afforded him. The day following he left the neighborhood free from disease. I have never seen any man suffer more from Rheumatism than did this man on my first visit, and am sure I never have seen one as quickly relieved. In regard to the modus operandi of the Cit. Acid, it has been supposed to act by converting uric acid into urea and carbonic acid ; the retained uric acid being looked upon as the cause of disease. How this may be I will not say, but that under the use of the acid the pulse is reduced and the flow of urine increased, is evident ; and above all, whereas my patients suffered great pain previous to the use of the acid, after its use, they were speedily relieved. —*Medical Reporter.*

